# Fifteen Cases of “Cerebro-Spinal Meningitis”

**Published:** 1867-03

**Authors:** Chas. M. Clark

**Affiliations:** Late Surgeon 29th Ill. Vol. Infantry, and Chief Operating Surgeon 24th Army Corps


***Fifteen Cases of "Cerebro-Spinal Meningitis," and their Post Mortem Appearances.*** Presented by CHAS. M. CLARK, M.D., late Surgeon 29th Ill. Vol. Infantry, and Chief Operating Surgeon 24th Army Corps.

*Continued from page 13.*

CASE X. Private John Hughes, Co. G, 158th New York Vols. American, aged 24. This man was admitted to Hospital December 22d, 1864. No previous history of the case obtained.

*Symptoms on Admission.*—Face flushed; respiration hurried, and talks incoherently—cannot be roused up. Pulse 120 and full; skin hot and dry; tongue slightly coated; some disposition to tonic spasm.

*Treatment.*—Styptic pediluvium and acetate potass.

*Progress of Case.—Dec. 23.* Continues unconscious; some spasm of flexor muscles. Strong enemata given him, combining antispasmodic. Pupil of eye contracted; is semi-delirious. Medicine seems to do no good.

*Dec. 24.* Died at 10 o'clock A.M., without any perceptible change being noticed in his case.

SECTIO CADAV. SEVENTEEN HOURS AFTER DEATH.

Body slightly emaciated, but presents good muscular development. Rigor mortis well marked. On removal of the calvarium, the membranes of the brain present a highly congested appearance. On removal of the brain, a turbid serum, to the amount of ℥iss., was found in the occipital fossa containing pus corpuscles. The cerebellum was intensely congested, and at its base the sub-arachnoid cavity was covered with a soft yellow exudation presenting every appearance of purulent matter. This was also found in spots over the whole cerebrum. On the left hemisphere and near the longitudinal sinus the arachnoid was raised from the brain by a collection or turbid serum, about ʒss in quantity. Cerebellum soft and pultaceous. Each of the lateral ventricles contained ʒj. of milky serum. Spinal cord congested throughout its length, but no exudations were apparent.

*Chest.*—Organs were normal.

*Abdomen.*—Nothing strictly abnormal could be discovered, except slight enlargement of the liver. Small intestines filled with fæcal matter. Bladder partly filled.

CASE XI. H. Manshur, private, Co. E, 2d N.H. Vol. Infantry. Admitted to hospital February 6th, 1865. This man had been sick in the regimental hospital two weeks prior to his entry; had chill followed by fever; also had diarrhœa. On the day of his admission he was delirious—pupils contracted; tongue dry and discolored; respiration hurried; pulse 130.

*Treatment.*—Pulv. Doveri and quinine given him; also milk punch, together with application of cold to the head and sinapisms to the feet. Catheter passed and small quantity of urine drawn off.

*Progress of Case.—Feb'y 7.* Continues delirious; appears to be suffering greatly; had several involuntary dejections from bowels during the night; some spasm of the muscles and disposition to trismus. Opium, quinine, and milk punch ordered given during the day.

*Feb'y 8.* No change in symptoms, except that he is more quiet. Continue opii. and quinine. Applied blister to back of neck.

*Feb'y 9.* Involuntary dejections continue; pupils of eyes dilated; well-marked tetanic spasm—cannot force open the jaws—well marked opisthotonos; abdominal muscles hard and rigid; some appearance of petechiæ over the body; hands and feet purplish in color and very cold. Medicine does no good.

*Feb'y 10.*. Died this morning.

AUTOPSY TEN HOURS AFTER DEATH.

*Brain.*—Upon removing the calvarium, the membranes of the brain appeared extended and puffed out with fluid, except the pia mater, which was closely adherent to the brain; convolutions of the cerebrum were covered with lymph, and in some places there were large patches of pus; ℨj. of flaky serum found in each of the lateral ventricles; substance of brain much softened. The cerebellum presented the same appearances. Membranes of the cord infiltrated with serum.

*Chest.*—Left Lung:—Old adhesions of apex very firm and hard, and some tubercular deposit. Right lung found normal. Heart normal; but there was an anomalous distribution of the vessels leading from it, there being no arteria innominata, the right subclavian and right carotid proceeding directly from the arch of the aorta, with a space of nearly one inch between their origins.

*Abdomen.*—Liver slightly enlarged; gall-bladder distended. Spleen enlarged about one-third, and had an extra lobe growing from it. Kidneys, as healthy as are generally found.

*Intestines.*—Colon distended; descending colon firmly bound down by adhesions old in character. Small intestines impacted with fæces; patches of ulceration found throughout the whole extent of the ileum; well marked appearance of inflammation over stomach and peritoneum generally; bladder partly filled.

CASE XII. F. R. Spillen, private, Co. H, 199th Penn. Vols. American, aged 26 years. Admitted to hospital February 26th. Previous history not known. At the time of his entrance had high fever; pulse 120; tongue furred; skin dry and hot; respiration hurried, with considerable cough; eyes injected and watery.

*Treatment.*—Mild cathartic given; surface of body sponged over with tepid water; 10 grs. pulv. Doveri and nit. potass. given.

*Progress of Case.—Feb'y 27.* Passed comfortable night; bowels moved freely; eruption noticed over whole surface of body; complains of severe pain in head, and states that his lungs are very sore. Cough continues. 2 grs. opii. and 10 nit. potass. ordered given every four hours. Ice applied to head.

*Feb. 28.* No improvement in the case. Urine passes naturally, but scant and high colored. Same treatment continued, with addition of milk punch every hour.

*Feb'y 29.* Rapidly failing. Some delirium and jactitation.

*March 1.* Died.

AUTOPSY 24 HOURS AFTER DEATH.

Body emaciated. Rigor mortis well marked.

*Brain.*—On removal of the brain from the skull, ℥ij. of serum was found in the occipital fossa; great effusion underneath the arachnoid, which was considerably thickened; pia mater extensively congested with both arterial and venous blood; lymph found in abundance over whole surface of cerebrum and cerebellum, but no pus. No serum found in the ventricles; brain tissue very much softened. Spinal cord not examined.

*Chest.*—Lungs found in normal condition. Heart:—Considerable effusion found within the pericardium; right auricle and ventricle filled with coagulated blood; large fibrinous clot found in left ventricle; left auricle one-third smaller than normal.

*Abdomen.*—Liver normal; gall bladder distended. Kidneys apparently healthy. Spleen atrophied; fully two-thirds smaller than usual.

*Intestines.*—Congested and spotted; glands of the mesentery enlarged; ℥iv. of fluid found in the abdominal cavity. Bladder fully two-thirds smaller than normal; found empty, and its coats greatly thickened.

Case XIII. G. W. Bean. Private Co. C, 9th Maine Vols. American, aged 24 years. Admitted to hospital January 16th, 1865, with what was considered a well marked eruption of rubeola; slight fever; pulse 98; severe cough, with pneumonic expectoration; severe pain in right side, and considerable dysponœa; tongue red and dry. Eruption had made its appearance three days before his *entree.* Made complaint of severe pain in his head and down his back. Pupils of eyes natural.

The treatment in this case was not given me by the Surgeon on duty, and all that is known concerning the progress of the disease is, that the man became delirious on the third day after his entrance, and continued insensible until the date of death, which occurred January 31st, at 3:30 o'clock A.M.

AUTOPSY, 2 O'CLOCK P.M.

Body greatly emaciated; rigor mortis well marked.

*Brain.*—Membranes of the brain greatly congested and distended with serum; arachnoid thickened; patches of lymph over surface of cerebrum and cerebellum. No effusion found in the ventricles; some little pus found over tract of optic commissure. Membranes of the cord infiltrated. Brain tissue softened.

*Chest.*—Right Lung:—Old and very firm adhesions binding down the whole lung to pleura-costalis. Upper lobe hepatized (red), and a portion of lower lobe. Left lung slightly congested, and pus found in the bronchiæ. No effusion in chest. Heart:—℥j. of serum in pericardium; otherwise normal. Liver hypertrophied one-third and mottled. Gall bladder partially empty. Spleen normal. Kidneys slightly congested. Stomach distended with gas.

*Intestines.*—Glands of mesentery enlarged; otherwise normal.

CASE XIV. James Kirkpatrick. Private Co. C, 199th Pa. Vols. Admitted to hospital January 20th, 1865. Symptoms on admission, treatment and progress of the disease not obtained. The man died February 2d, 1865.

AUTOPSY TWELVE HOURS AFTER DEATH.

*Brain.*—Considerable effusion under the membranes, and dura mater in a high state of congestion; arachnoid thickened. Over right hemisphere of cerebrum there was a large deposition of lymph; pia mater thickened, and adherent in some places to the brain. Convolutions of brain not well marked; pus found in small quantity over the optic tract; ventricles partly filled with turbid serum; brain tissue soft and easily broken down; spinal cord much congested.

*Chest.*—Left Lung:—Commencing hepatization in upper lobe, and some adhesion to pleura-costalis. Other portions of lung apparently healthy. Right Lung:—Old adhesions bending down the lung firmly throughout the whole extent. Heart:—℥iij. of serum in pericardium; veins of heart greatly enlarged; substance flabby and slightly hypertrophied.

*Abdome* .—Liver one-third larger than normal. Gall bladder empty. Spleen normal. Kidneys healthy as usually found. Stomach:—Some signs of peritonitis. Intestines:—Considerable effusion found in cavity; whole tract congested; vessels of the omentum greatly engorged and distended. Bladder found partially empty.

CASE XV. Samuel Farnsworth, Private Co. H, 10th N. H. Vols. Admitted to hospital on the evening of December 21st, with well-marked fever; pulse 120; tongue thickly coated; skin dry and burning; difficult respiration; diarrhœa, with involuntary discharges; severe cough, with free expectoration; constant pain ascribed to back of head and neck; urine voided freely, but of dark color.

*Treatment.*—Warm bath; pulv. Doveri.

*Progress of Case.—Dec. 22.* Is feeling somewhat better, but inclined to be restless. Mind rather obtuse; pupils natural; pain in back continues; discharges from bowels quite frequent. Ice applied to head; sinapism to ankles and chest; spr. Mindererus given every hour; quinine every four hours.

*Dec. 23.* It is apparent that medication will do no good. Some delirium; surface of body cold. Milk punch given every hour.

*Dec. 24.* Died comatose at 10 P.M.

SECTIO CADAV. TWELVE HOURS AFTER DEATH.

*Brain.*—Dura mater intensely congested, with some effusion underneath it; some lymph deposited over the lobes of the cerebellum; ventricles dry; brain tissue softened; meninges of cord congested through cervical region.

The examination of the chest showed double pleuro-pneumonia; lower lobe of left lung hepatized and slightly adherent to pleura-costalis. A grayish purulent matter exuded upon incision into the lung. ʒiv. of light straw-colored serum found in left cavity of chest. Right lung slightly adherent to the pleura-costalis, with red hepatization throughout in advanced stage. Heart normal. Liver, spleen and kidneys apparently healthy. Stomach and small intestines distended with gas. Intestines contained but little fæcal matter. Mucous membrane congested throughout its whole extent, but no ulcerations found, except in the cœcum and lower part of the rectum, the mucous lining of these parts presenting a dark mahogany color.

The body of this man was greatly emaciated, and presented a peculiar dirty ash color.

REMARKS.

At the time this disease, which I have denominated *Cerebro-Spinal Meningitis*, made its appearance, the troops were comfortably quartered about three miles back from Varina Landing on the James River. The country was high and rolling, and heavily timbered, the soil being a mixture of sand and clay. The season had been remarkably wet, and intermittent, remittent and typho-malarial fevers were prevailing extensively. The men were also greatly and continuously exposed to fatigue and excitement, there being almost constant calls upon them for duty in reconnoitering, constructing earth-works, and fighting the enemy; and it is but fair to presume that the continual hardship and excitement that the men endured, and withal, considering that they were in a decidedly miasmatic region, were conditions that undoubtedly predisposed to attacks of this malady. Before going farther, I desire to remark that I consider the disease in mention an intensified form of typho-malarial fever, the determination being to the brain and its membranes. I do not know that I have sufficient facts to base this opinion upon; but my experience with the disease while in the army has led to this belief. In the first place, the disease seldom manifests itself, except in miasmatic regions; secondly, the premonitory symptoms are such as generally usher in typho-malarial fever, such as—feeling of general lassitude; chill, followed by high febrile action; pain in the head and bones generally; soreness of the flesh; tinnitus aurium; vomiting of bilious matters; brown tongue, with sordes; tenderness of the abdomen, etc. Thirdly, in the second stage, internal congestion, with pallid, cold surface; sometimes an eruption; delirium or coma; tonic or tetanic spasm, etc., until death closes the drama.

Fourthly. The analogy is, I think, confirmed in the *post mortem* appearances. In making examination of cases that have succumbed to typho-malarial fever, I have found the membranes of the brain congested, thickened, and distended with serum, exudations of lymph in some places, and softening of the medulla; heart often hypertrophied, and pericardium containing serum; pleura throwing out lymph; liver and spleen engorged; gall-bladder sometimes distended, sometimes empty; intestines congested; glands of mesentery enlarged, and sometimes well-marked ulceration.

Fifthly. In the treatment, tonics, stimulants, revulsives and counter-irritants are the only remedies that seem to do good.

It is a matter of regret that a full record of the previous history in each of the foregoing cases was not obtained, as it would not only have enhanced the interest attaching to them, but would have been more satisfactory in a practical point of view. The majority of the cases treated were not sent to hospital until the disease had made fatal progress, and there was no hope of saving the life of the patient. Some of the cases are also incomplete in the matter of giving full symptoms on admission, treatment, and progress of the disease, from the fact that the medical officers in immediate attendance neglected to note them down. In regard to the treatment given the cases that came under my notice, I must admit that it was decidedly speculative. The many agents of the materia medica, considered as applicable to the disease, were tried time and again, but without materially changing its condition or character. The remedies chiefly depended upon were full doses of quinine and opium, together with the free use of stimulants (in the form of milk punch) and counter-irritants. The fatality attending the disease was remarkable. I know of only two cases that manifested strong symptoms of the malady that recovered, and they were undoubtedly cases of mistaken diagnosis, or else extremely mild in character. I do not believe that the disease was due to epidemic influence; for, had it been, there would have occurred a greater number of cases, out of the large number of men constituting the 24th Army Corps, many of whom were equally as predisposed, by reason of habits, temperament, diseased condition, etc., as those who were attacked.

